# Differences in brain functioning between obsessive-compulsive disorder and generalized anxiety disorder: a clinical study using functional near-infrared spectroscopy

**DOI:** 10.3389/fpsyt.2026.1757526

**Published:** 2026-03-18

**Authors:** Luyao Wang, Yuan Tian, Yiping Luo, Qiao Lu, Genwen Sun, Xia Deng, Xu Zhang

**Affiliations:** Sichuan Provincial Center for Mental Health, Sichuan Provincial People’s Hospital, School of Medicine, University of Electronic Science and Technology of China, Chengdu, China

**Keywords:** brain functioning, functional near-infrared spectroscopy (fNIRS), generalized anxiety disorder (GAD), obsessive-compulsive disorder(OCD), verbal fluency task

## Abstract

Generalized anxiety disorder (GAD) and obsessive-compulsive disorder (OCD) share common epidemiological and clinical features, but their shared neurobiological basis remains unclear. This study was designed to examine similarities and differences in brain functioning between GAD and OCD using functional near-infrared spectroscopy (fNIRS). 31 patients diagnosed with GAD, 31 with OCD, and 31 healthy controls (HCs) participated in the study. A 53-channel fNIRS system was used to measure changes in oxygenated hemoglobin ([oxy-Hb]) concentration during a verbal fluency task. Five regions of interest (ROIs) were defined: premotor cortex/supplementary motor area (SMA), Broca’s area, dorsolateral prefrontal cortex (DLPFC), frontal eye fields (FEF), and frontopolar area (FPA). Using channel-based and ROI-based analysis strategies, [oxy-Hb] changes across groups were compared via one-way ANOVA and *post hoc* tests. HCs exhibited significantly greater brain activation in multiple regions (DLPFC, prefrontal cortex [PFC], Broca’s area) than both patient groups. *Post hoc* analysis revealed that GAD patients showed higher activation in the left DLPFC and left FPA compared to OCD patients. Correlation analysis indicated that activation in the left Broca’s area was significantly negatively correlated with obsessive-compulsive symptoms. Receiver operating characteristic (ROC) curve analysis demonstrated that the right FPA [oxy-Hb] value optimally discriminated patient groups (OCD and GAD) from HCs (optimal cutoff: 92.94505; sensitivity: 0.643; specificity: 0.816; AUC: 0.769 [95% CI: 0.661–0.877; *p* < 0.001]). These findings suggest that while GAD and OCD share overlapping neurofunctional alterations, OCD patients showed more marked deficits in the left DLPFC and left FPA.

## 1 Introduction

Accounting for the highest prevalence among mental disorders in the general population, anxiety disorders have a lifetime rate of 34% (1). Among these, GAD is particularly common, with a lifetime prevalence of 6.2% (2, 3), and significantly impairs quality of life and functioning. OCD formerly categorized as an anxiety disorder prior to its separation in DSM-5—has a lifetime prevalence of 2–3% (4). Notably, over 25% of individuals with OCD have a co-occurring anxiety disorder (5). GAD is characterized by persistent, uncontrollable worry, tension, or fear (6), while OCD manifests through recurrent, intrusive thoughts (obsessions) and ritualistic behaviors (compulsions), characteristically paired by anxiety (7). Although both disorders involve anxiety, their anxiety sources and coping mechanisms differ markedly: Anxiety of GAD patients is often free-floating, lacking specific triggers, with patients typically utilizing avoidance or somatization for relief. Anxiety of OCD patients is primarily triggered by obsessions, with compulsions employed to alleviate distress. This phenomenological overlap frequently complicates differential diagnosis in early illness stages.

Both Generalized Anxiety Disorder (GAD) and Obsessive-Compulsive Disorder (OCD) are thought to involve dysregulation of the prefrontal cortex; however, current neurobiological models propose that their predominant neural circuits may differ. Evidence suggests that the pathophysiological basis of GAD is more closely linked to an imbalance in the “fear/anxiety generation circuit” (such as the amygdala-prefrontal circuit), often characterized by hyperactivation in regions including the amygdala and insula (8), alongside evidence of reduced inhibitory control of the amygdala by the ventromedial prefrontal cortex (vmPFC) (9). In contrast, a leading model posits that the core dysfunction in OCD lies within the corticostriatal-thalamo-cortical (CSTC) circuit (10), particularly implicating pathways related to behavioral control and habit formation. Consistent with this model, structural neuroimaging studies have revealed abnormalities in gray matter volume in brain regions associated with the CSTC circuit (e.g., orbitofrontal cortex, anterior cingulate cortex, thalamus) in individuals with OCD (11). Despite these proposed distinctions in underlying circuitry, both disorders are characterized by impairments in prefrontal cortex-mediated executive functions (12), such as cognitive control and response inhibition, which are essential for adaptive behavior.

In terms of network connectivity, studies have associated GAD with hyperconnectivity within the default mode network and aberrant interactions with the salience network (13)-,dysfunctions in which OCD is also implicated-,reflecting the persistent and intrusive self-referential worries characteristic of the disorder. Additionally, research on OCD has consistently reported dysfunctions within the cognitive control network and the salience network, as well as in their interactions with the CSTC circuit (14), mirroring the difficulties in behavioral inhibition and habit termination observed in the condition. Thus, prior literature indicates both overlapping and dissociable patterns of functional connectivity alterations in GAD and OCD.

As a non-invasive optical neuroimaging technique, fNIRS measures cortical hemodynamic changes via oxygenated hemoglobin [oxy-Hb] and deoxygenated hemoglobin [deoxy-Hb] levels. Its high tolerance to motion artifacts and relative ecological validity make it particularly suitable for studying clinical populations, including individuals with anxiety-related disorders who may experience restlessness during scanning. Crucially, fNIRS provides excellent sensitivity to the prefrontal cortex (15), the key region implicated in the pathophysiology of both OCD and GAD, thereby offering a direct means to test hypotheses regarding disorder-specific and shared prefrontal dysfunction. The Verbal Fluency Task (VFT) was selected as the cognitive paradigm because it robustly engages prefrontal executive functions—including cognitive control, working memory, and inhibitory processing—that are consistently reported to be impaired in both OCD and GAD. By imposing a time-pressured demand on verbal generation and self-monitoring, the VFT can effectively challenge the very cognitive control systems hypothesized to be dysregulated in these disorders, thereby serving as a potent probe to reveal disorder-specific cortical activation patterns (16).

Consistent evidence indicates that reduced prefrontal [oxy-Hb] during VFT performance is a replicable finding in several major psychiatric disorders, most notably major depressive disorder and schizophrenia, when compared to HCs (17, 18). However, disorder-specific patterns emerge: Liao et al. (52-channel system; N = 70 OCD vs. 70 HCs) reported elevated [oxy-Hb] changes across most prefrontal and temporal regions in OCD patients versus HCs, with these changes significantly correlating with obsessive-compulsive symptom severity (19). Hu et al. (N = 51 GAD vs. 47 HCs) identified hypoactivation specifically in the left ventrolateral prefrontal cortex (VLPFC) and left DLPFC (20). Collectively, these findings suggest that fNIRS-derived hemodynamic responses during cognitively demanding tasks like the VFT may serve as quantifiable indices of frontally mediated dysfunction, potentially correlating with clinical symptom burden. Critically, no existing study has directly compared disorder-specific fNIRS activation patterns between OCD and GAD during VFT performance. Filling this direct comparison gap is critical for advancing a more nuanced understanding of anxiety-related disorders. Specifically, it can help disentangle whether observed cortical hypoactivation represents a transdiagnostic marker of general cognitive control impairment common to both OCD and GAD, or whether it comprises disorder-specific patterns that could inform more precise pathophysiological models. Furthermore, clarifying these activation signatures is a necessary step toward evaluating the potential of fNIRS metrics as auxiliary tools for differential diagnosis or treatment target identification.

Given that (a) both OCD and GAD involve prefrontal executive dysfunction, yet may arise from distinct neurocircuitry models, and (b) fNIRS activation during the VFT is a sensitive probe of such dysfunction and may relate to clinical severity, a direct comparison between these disorders is warranted to address two key questions: First, to what extent do OCD and GAD share common patterns of cortical hypoactivation during cognitive challenge, reflecting a transdiagnostic deficit, and to what extent do they differ? Second, are these disorder-specific or shared activation patterns meaningfully correlated with the severity of core clinical symptoms?

Therefore, this study compared activation patterns across brain regions in OCD, GAD, and HCs during a VFT using 53-channel fNIRS, while examining relationships between regional activation and clinical symptoms. We proposed two hypotheses: 1. OCD and GAD patients demonstrate partially shared regional hypoactivation, with OCD showing more severe impairment than GAD; 2. Regional hypoactivation shows significant correlations with symptom severity of GAD and OCD.

## 2 Materials and methods

### 2.1 Participants

Clinical data were collected from 31 OCD patients and 31 GAD patients hospitalized in the Department of Psychosomatic Medicine at Sichuan Provincial People’s Hospital between June 2023 and June 2024. During the same period, 31HCs were recruited with gender, age, and education matching. Demographic characteristics (gender, age, education years) showed no significant differences between groups (**Table 1**).

**Table 1:** Coverage of brain regions by 53 fNIRS channels.

Brodmann Areas (BA)	Channels
Premotor Cortex and Supplementary Motor Area (SMA)	CH01 CH04 CH10 CH40 CH47 CH52
Broca’s area	CH02 CH03 CH05 CH07 CH08 CH13 CH46 CH49 CH50 CH51 CH53
Dorsolateral Prefrontal cortex (DLPFC)	CH06 CH09 CH11 CH14 CH17 CH18 CH20 CH25 CH31 CH32 CH34 CH39 CH42 CH45 CH48
Frontal Eye Fields (FEF)	CH12 CH24 CH26 CH38
Frontal Pole Area (FPA)	CH15 CH16 CH19 CH21 CH22 CH23 CH27 CH28 CH29 CH30 CH33 CH35 CH36 CH37 CH41 CH43

Inclusion criteria for patient groups: (a) DSM-5 diagnosis of OCD or GAD confirmed by ≥1 senior psychiatrist (deputy chief physician or higher); (b) Age 18–65 years; (c) Right-handedness (Edinburgh Handedness Inventory); (d) Completion of at least 6 years of formal education; (e) No clinically significant structural abnormalities on cranial MRI scans.

Exclusion criteria (all groups): (a) Comorbid DSM-5 psychiatric disorders (schizophrenia, depression), neurological disorders, organic mental disorders, or traumatic brain injury; (b) Left-handedness; (c) Pregnancy or lactation; (d) Substance/alcohol dependence; (e) Contraindications for fNIRS.

All participants provided written informed consent following comprehensive study disclosure. Ethical approval was granted by the Sichuan Provincial People’s Hospital Ethics Committee. (Ethical Approval No. 331 [2022]).

### 2.2 Clinical scales

The Yale-Brown Obsessive Compulsive Scale (Y-BOCS), developed by Goodman et al. (1989) (21), assesses obsessive-compulsive symptom severity (22). This 10-item instrument comprises items 1–5 measuring obsession severity and items 6–10 measuring compulsion severity, using a 5-point scale (0–4) per item. Severity is classified by total scores as: subclinical (1–7), mild (8–15), moderate (16–23), severe (24–30), or extreme (32–40). The Chinese adaptation showed excellent internal consistency (α = 0.81) and high temporal stability (test-retest r = 0.83) (23), confirming strong reliability and validity.

### 2.3 Experimental paradigm

**Figure 1:**
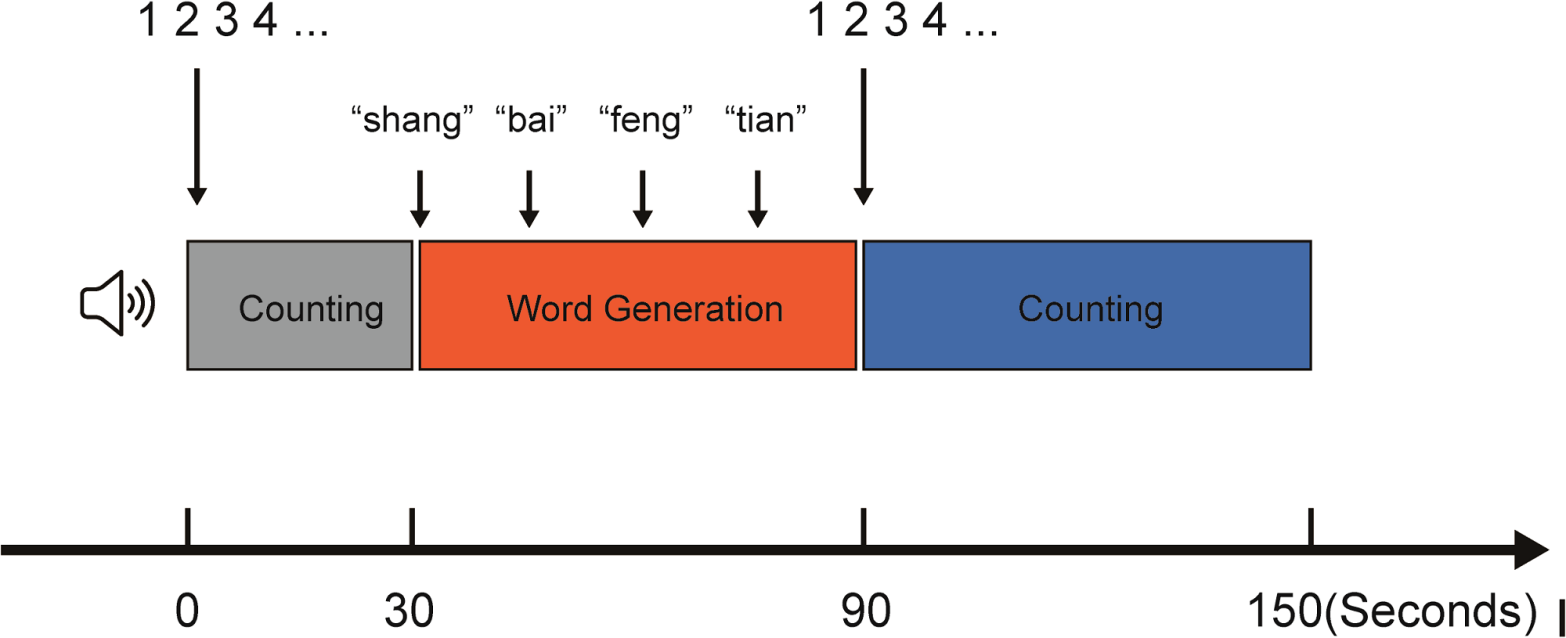
Flowchart of the VFT paradigm.

This study employed a VFT comprising three phases (1): Pre-experimental phase: participants counted 1–16 following auditory prompts (30 s) (2); Task phase: participants performed a semantic (category) fluency task by generating words semantically related to each of four sequentially presented Chinese characters (上, 白, 风, 天), each presented for 15 s in fixed order (3); Recovery phase: participants counted 1–33 following auditory prompts (60 s). fNIRS data were synchronously recorded throughout the experiment (paradigm flow: **Figure 1**).

### 2.4 fNIRS data acquisition

**Figure 2:**
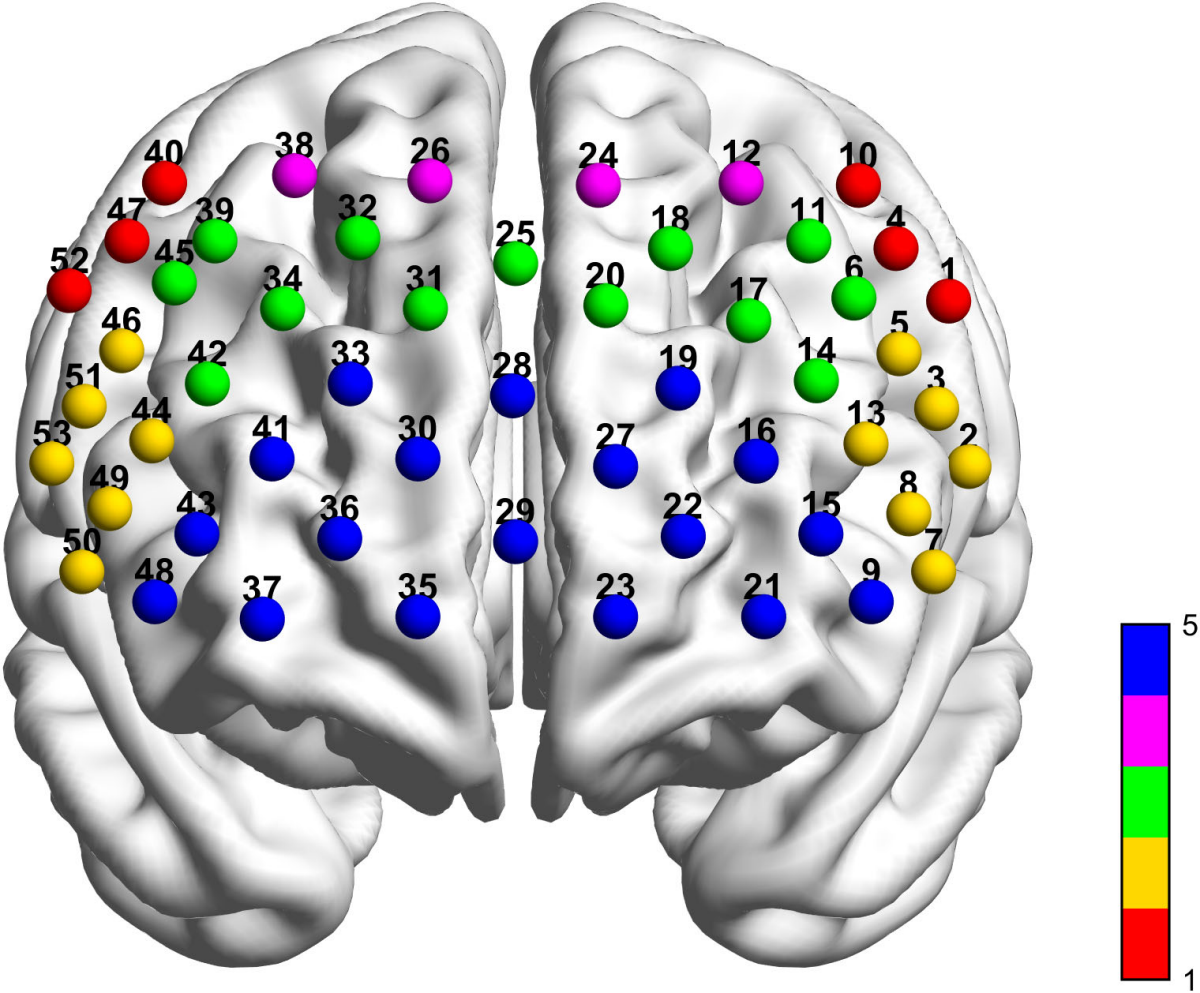
53-channel alignment diagram (The red sphere represents the Premotor Cortex and Supplementary Motor Area. The yellow sphere represents the Broca’s area. The green sphere represents the Dorsolateral Prefrontal cortex. The purple sphere represents the Frontal Eye Fields. The blue sphere represents the Frontal Pole Area.).

Participants completed tasks in a sound-attenuated room and instructed to minimize head movement during testing. fNIRS data acquisition employed a 53-channel system (BS-5000L, Wuhan Zilian Hongkang Co., Ltd.) covering frontal, partial temporal, and parietal cortices. The montage comprised 16 sources and 16 detectors arranged in a grid (3 cm source-detector distance), with photopole 9 positioned at the FPz reference point. Optodes were symmetrically distributed along the T3-T4 axis with posterior fixation (**Figure 2**). The system utilized dual-wavelength near-infrared light (690 nm and 830 nm) for hemodynamic detection.

Region of interest (ROI) partitioning followed Brodmann-based parcellation. The 53 channels covered five cortical regions: premotor cortex and supplementary motor area (SMA), Broca’s area, dorsolateral prefrontal cortex (DLPFC), frontal eye fields (FEF), and frontopolar area (FPA), with specific channel assignments detailed in **Table 1**.

### 2.5 Data processing flow

Data processing was performed using HOMER2 software with the following pipeline (1): downsampling to 20 Hz (2); removal of channels exceeding quality thresholds (coefficient of variation >25%) (3); computation of [oxy-Hb] changes via the modified Beer-Lambert law (MBLL) (4); 5-s moving window averaging, with trimming of initial 7.5 s and final 2.5 s (5); baseline correction using rotational translation, where a linear baseline was constructed from pre-stimulus (-10 to -5 s) and post-task data;

### 2.6 Statistical analyses

#### 2.6.1 Analysis of demographic and clinical variables

One-way analysis of variance (ANOVA) was used to compare age and years of education across the obsessive-compulsive disorder (OCD), generalized anxiety disorder (GAD), and healthy control (HC) groups. Chi-square tests were used to compare gender distribution. The threshold for statistical significance was set at *p* < 0.05.

#### 2.6.2 fNIRS data analysis strategy and ROI aggregation

All subsequent analyses focused on [oxy-Hb] due to its superior signal-to-noise ratio and more direct reflection of task-related cortical activation (24). Analyses were conducted at two levels (1): channel-wise analysis for exploratory topographic mapping, and (2) region of interest (ROI) analysis based on *a priori* parcellation (see **Table 1** for the complete channel-to-ROI mapping). Specifically, channels were grouped into anatomical ROIs (e.g., dorsolateral prefrontal cortex, temporo-parietal junction) based on standard brain atlas coordinates. For each participant and channel, five hemodynamic parameters were extracted: temporal integral, center of gravity, slope (scaled by 10^4^ to prevent computational instability), peak value, and mean amplitude. For each participant and ROI, the final value for each parameter was calculated as the arithmetic mean across all channels assigned to that ROI. Additionally, the mean [oxy-Hb] activation change across all 53 channels was quantified as the difference between the task-phase and pre-task-phase mean values for exploratory purposes.

#### 2.6.3 Hypothesis testing for group differences

The five ROIs (DLPFC, FPA, Broca’s area, SMA, and FEF) were defined based on the spatial coverage of our fNIRS montage. Based on prior literature, we formulated differential predictions for these regions. Specifically, we hypothesized that the DLPFC, FPA, and Broca’s area—given their established roles in cognitive control, internally guided cognition, and verbal inhibition—would show reduced [oxy-Hb] activation in both patient groups compared to HCs, with OCD exhibiting more pronounced reductions than GAD, particularly in the DLPFC. In contrast, the SMA and FEF are primarily involved in motor preparation and oculomotor control rather than the cognitive–affective processes central to our hypotheses; therefore, analyses in these regions were considered exploratory, and no directional predictions were made.

To test these predictions, we applied a sequential statistical approach for each of the five hemodynamic parameters (integral, center of gravity, slope, peak, and mean) extracted from the [oxy-Hb] time series. First, Levene’s test assessed homogeneity of variances. Based on its outcome, a standard one-way ANOVA was conducted when variances were homogeneous (p ≥ 0.05), whereas Welch’s ANOVA was used when variances were heterogeneous (p < 0.05). For parameters with a significant omnibus test result (p < 0.05), *post-hoc* pairwise comparisons were then performed: the LSD test was applied following a standard ANOVA, and the Tamhane’s T2 test was applied following Welch’s ANOVA. To control for multiple pairwise comparisons within each parameter, the false discovery rate (FDR) correction was applied separately to the three *post-hoc* p-values for that parameter. Exploratory channel-wise results were visualized in topographic plots.

#### 2.6.4 Exploratory evaluation of diagnostic utility

Receiver operating characteristic (ROC) analysis was conducted to explore the potential of the fNIRS-derived mean [oxy-Hb] activation change across all channels in differentiating the combined patient group (GAD+OCD) from healthy controls (HC). The area under the curve (AUC) was calculated. For analyses with significant discriminatory power (AUC > 0.5), the optimal cutoff value was determined by maximizing the Youden index (J = sensitivity + specificity - 1).

#### 2.6.5 Software

All statistical analyses were performed using SPSS software (version 26.0).

## 3 Results

### 3.1 Demographic characteristics of participants

Demographic characteristics were shown in **Table 2**. Gender, age, and education level did not differ significantly across the three groups.

**Table 2:** Demographic characteristics of patients and normal controls.

Demographics	OCD	GAD	HC	F/χ^2^	P-value
Gender(female/male)	17/14	13/18	18/13	1.808	0.405
Age(yr)	31.45 ± 14.41	36.84 ± 11.66	36.84 ± 14.45	1.634	0.201
educational level(yr)	13.32 ± 2.79	13.74 ± 3.08	14.97 ± 2.89	2.656	0.076

### 3.2 Group comparisons of oxy-Hb and deoxy-Hb changes per channel

**Figure 3:**
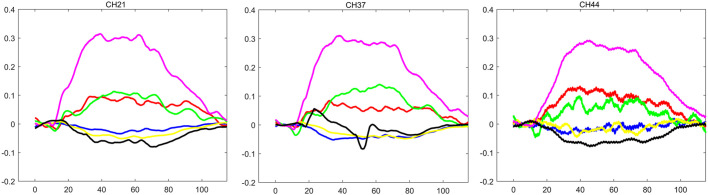
Changes in HbO and HbR of 3 channels during the VFT task in 3 groups (The vertical axis shows the change in concentration for [oxy-Hb] and [deoxy-Hb]. The horizontal axis shows time. The Red line represent fluctuations in [oxy-Hb] during VFT in OCD. The blue line represent fluctuations in [deoxy-Hb] during VFT in OCD.The green line represent fluctuations in [oxy-Hb] during VFT in GAD. The yellow line represent fluctuations in [deoxy-Hb] during VFT in GAD.The purple line represent fluctuations in [oxy-Hb] during VFT in HCs. The black line represent fluctuations in [deoxy-Hb] during VFT in HCs).

**Figure 4:**
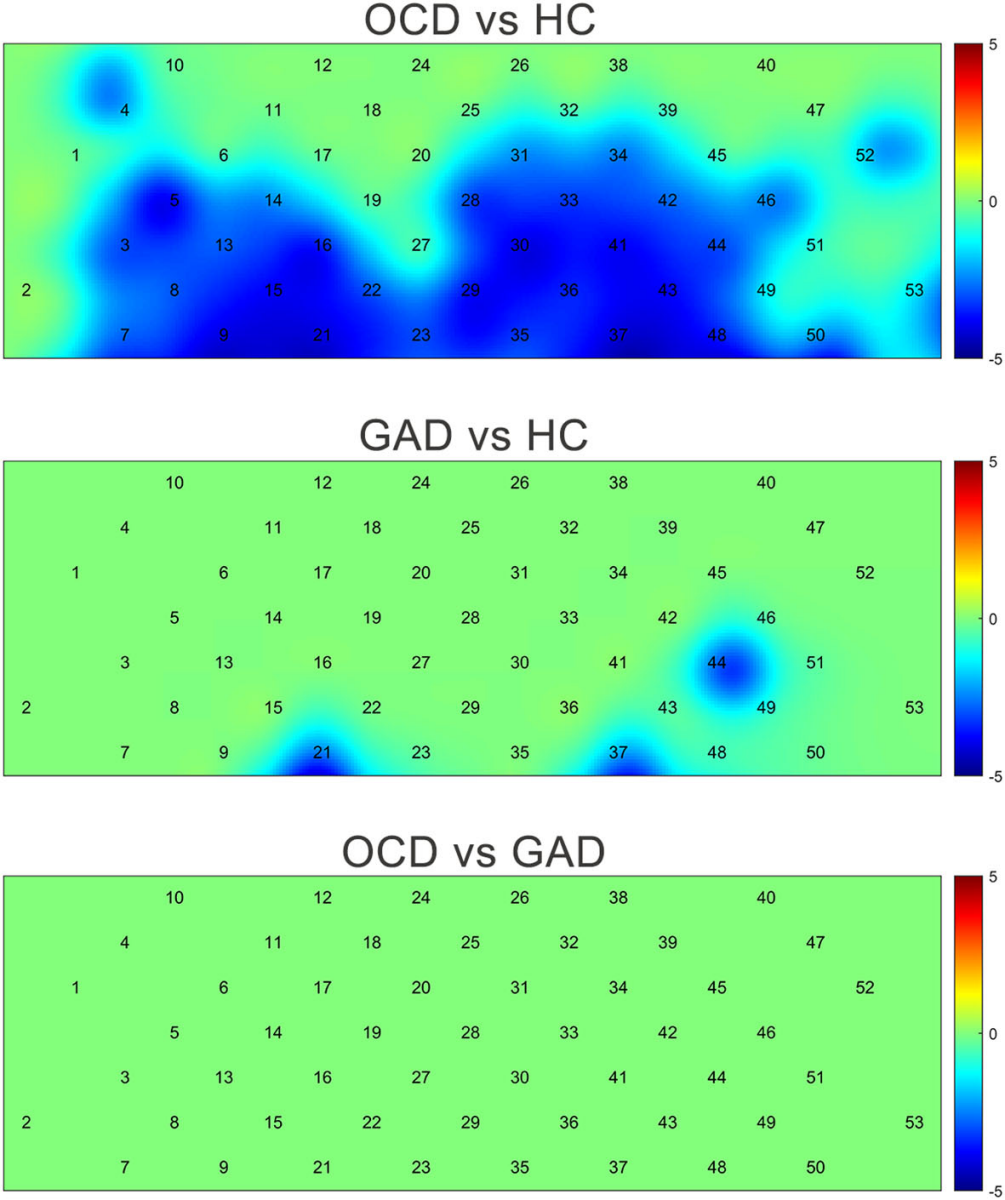
Comparison of [oxy-Hb] changes among three groups by each channel. (Color bar represent t values).

After FDR correction (*p* < 0.05), the OCD group showed significantly lower [oxy-Hb] concentrations than HCs in 32 channels (3–5, 7–9, 13–16, 21–23, 25–40). The GAD group showed significantly reduced [oxy-Hb] versus HC in 3 channels (21, 33, 37). No channels survived FDR correction for OCD-GAD comparisons. Critically, OCD and GAD shared three hypoactive channels relative to HC (21, 33, 37) (**Figure 3**), anatomically localized to the FPA (ch21, ch37) and Broca’s area (ch44) per Brodmann mapping (**Figure 4**).

### 3.3 Group comparison of fNIRS variables by region of interest

ROI-based comparisons revealed significant group differences in bilateral DLPFC, FPA, and Broca’s area (**Table 3**). Consistent with our hypothesis of more pronounced prefrontal deficits in OCD, p*Post hoc* analyses revealed that the integral value and mean value of left DLPFC, as well as the slope of left FPA, were significantly lower in OCD than in GAD (all p < 0.05).

**Table 3:** Comparison of fNIRS variables among three groups by region of interest.

Brain area	F	P	*Post hoc* analyses
Integral Value of Left DLPFC	5.638	0.005	GAD > OCD, HC > OCD
Integral Value of Right DLPFC	3.159	0.048	HC > OCD
Integral Value of Left FPA	9.293	0.000	HC > OCD, HC > GAD
Integral Value FPA	9.425	0.000	HC > OCD, HC >GAD
Integral Value of Left Broca	7.069	0.002	HC > OCD
Integral Value of Right Broca	5.566	0.006	HC > OCD
Slope of Left FPA	3.365	0.040	GAD > OCD
Mean of Left DLPFC	5.481	0.006	GAD >OCD,HC> OCD
Mean of Right DLPFC	3.265	0.044	HC >OCD
Mean of Left FPA	8.726	0.000	HC >OCD, HC > GAD
Mean of Right FPA	8.168	0.001	HC > OCD, HC > GAD
Mean of Left Broca	6.753	0.002	HC > OCD
Mean of Right Broca	6.072	0.004	HC > OCD, HC > GAD
Peak of Left DLPFC	5.174	0.008	HC >OCD
Peak of Left FPA	7.201	0.001	HC >OCD, HC > GAD
Peak of Right FPA	7.890	0.001	HC > OCD, HC > GAD
Peak of Left Broca’s	4.215	0.018	HC > OCD
Peak of Right Broca’s	3.872	0.025	HC > OCD

### 3.4 Correlation between fNIRS variables in ROIs of OCD and clinical assessments

Within the OCD group, fNIRS hemodynamic parameters in the left Broca’s area showed significant negative correlations with Y-BOCS scores (obsessions, compulsions, total). The integral value correlated negatively with Y-BOCS total (*r* = -0.45, *p* < 0.05) and compulsions (*r* = -0.48, *p* < 0.05). Peak were negatively correlations with Y-BOCS total (*r* = -0.51, *p* < 0.05) and compulsions (*r* = -0.56, *p* < 0.01). Mean value were negatively correlated with Y-BOCS total (*r* = -0.47, *p* < 0.05) and compulsions (*r* = -0.50, *p* < 0.05) (**Table 4**).

**Table 4:** Correlation between fNIRS variables in ROIs of OCD and clinical assessments (Non-significant correlations are not shown).

fNIRS variable	Y-BOCS total	Y-BOCS compulsions
Integral Value of Left Broca	-0.45*	-0.48*
Peak of Left Broca	-0.51*	-0.56**
Mean value Left Broca	-0.47*	-0.50*

### 3.5 ROC analysis for differentiating patients from healthy controls

**Figure 5:**
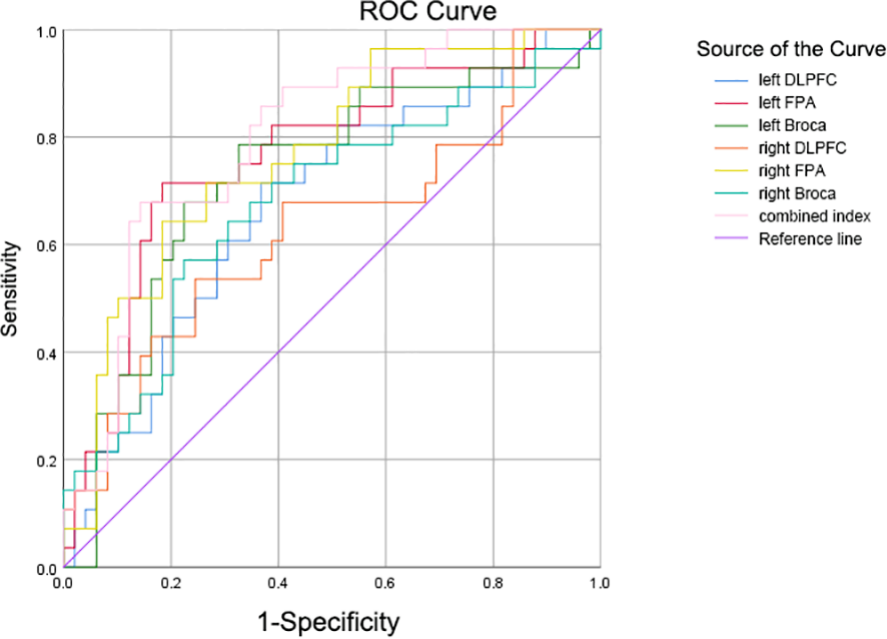
ROC for fNIRS features in the classification of patients with OCD and GAD vs HCs.

ROC analysis of all fNIRS variables showing significant differences among OCD, GAD, and HC groups revealed that integral values in five regions effectively distinguished combined OCD/GAD patients from HCs (*p* < 0.05): left DLPFC, left FPA, right FPA, left Broca’s area, and right Broca’s area (**Figure 5**). The right FPA integral value demonstrated optimal classification (AUC = 0.769, 95% CI: 0.661–0.877, *p* < 0.001), with a cutoff of 92.945 yielding sensitivity= 0.643 and specificity= 0.816 (**Table 5**). However, none of these parameters significantly differentiated OCD from GAD.

**Table 5:** Classification performance of fNIRS indicators.

fNIRS variable	Optimal cut-off value	Sensitivity	Specificity	AUC (95% CI)	P-value
Integral value of left DLPFC	36.2072	0.714	0.633	0.676 (0.551-0.800)	0.011
Integral value of right DLPFC	56.01945	0.536	0.755	0.633 (0.498-0.767)	0.054
Integral value of left FPA	82.85775	0.714	0.816	0.766 (0.653-0.879)	0.000
Integral value of right FPA	92.94505	0.643	0.816	0.769 (0.661-0.877)	0.000
Integral value of left Broca	71.60885	0.786	0.673	0.729 (0.607-0.851)	0.001
Integral value of right Broca	109.94865	0.571	0.776	0.680 (0.552-0.808)	0.009

## 4 Discussion

We believe this to be the first fNIRS investigation of brain function differences between OCD and GAD during VFT performance. Both channel- and region-of-interest (ROI) analyses yielded convergent results: relative to HCs, GAD showed significant hypoactivation in bilateral FPA and right Broca’s area, while OCD exhibited hypoactivation in bilateral DLPFC, bilateral FPA, and bilateral Broca’s area. Additionally, in OCD, reduced [oxy-Hb] activation in left Broca’s area correlated negatively with Y-BOCS compulsion severity. Integral values in DLPFC, FPA, and Broca’s areas effectively discriminated OCD/GAD patients from HCs. These findings confirm our hypotheses that both disorders share overlapping neurofunctional impairments—with more pronounced deficits in OCD—and that impairment severity correlates with clinical symptom severity. Critically, this pattern of shared and distinct deficits provides a neurofunctional basis for reconceptualizing these disorders through a dimensional and transdiagnostic lens, such as the Research Domain Criteria (RDoC) framework. This perspective shifts the focus from categorical diagnoses to underlying, continuous neurocognitive processes that may be dysregulated across diagnostic boundaries (41).

These observed patterns of shared and distinct prefrontal hypoactivation invite interpretation within contemporary, multi-system models of psychopathology. Specifically, the oxidative stress and neurotoxicity framework provides a compelling pathophysiological account, particularly for OCD (25, 26, 42). Crucially, such molecular and cellular disturbances are known to impair the neurovascular unit and the efficiency of neurovascular coupling—the physiological process underlying fNIRS signals (27). Oxidative stress is known to disrupt endothelial function and nitric oxide signaling, both of which are critical for neurovascular coupling (28). Consequently, the pronounced prefrontal hypoactivation observed in our OCD cohort may be reinterpreted not merely as a functional deficit, but as a hemodynamic correlate of upstream oxidative stress impacting cerebral metabolism and vascular responsivity. This integrative perspective aligns with transdiagnostic approaches by proposing shared biological vulnerabilities (41) (e.g., redox imbalance) that may manifest as overlapping functional impairments across disorders, while also providing a specific mechanistic hypothesis to explain the more severe deficits observed in OCD.

### 4.1 Both OCD and GAD exhibit hypoactivation in FPA and Broca’s area versus HCs

OCD and GAD exhibit similar neurofunctional alterations, with both disorders demonstrating hypoactivation in FPA and Broca’s area relative to HCs. Extensive prior research consistently reports prefrontal and temporal hypoactivation across both conditions, though with regional variations. OCD studies document broader hypoactivation patterns, including bilateral orbitofrontal cortex (OFC), inferior frontal gyrus (IFG), temporal gyri (TG) (43), inferior prefrontal cortex (IPFC), left superior temporal gyrus (STG), and bilateral middle temporal gyrus (MTG) (19). These discrepancies may reflect methodological differences in ROI selection and task design across studies. While prior work identified GAD hypoactivation primarily in left ventrolateral (VLPFC) and DLPFC (20, 29) using working memory tasks, our study found no significant DLPFC differences in GAD. This divergence may be attributable to differences in task paradigms: previous studies typically employed N-back tasks with higher working memory load, whereas our study used a verbal fluency task, which engages different cognitive processes and prefrontal subregions (30). Additionally, prior studies often defined ROIs based on *a priori* anatomical boundaries, while our approach used functionally defined ROIs derived from task-related activation, which may yield more sensitive or specific group comparisons (31). This findings underscores the need for standardized cognitive tasks and ROI definition methods in future research to facilitate cross-study comparisons. Although no NIR studies have directly identified functional changes in FPA in patients with GAD, a study comparing anxious and non-anxious depression found functional brain differences in FPA between the two group (32), while studies from imaging confirmed a significant association between FPA and anxiety mood (33). Beyond supporting phenomenological overlap between OCD and GAD, the common hypoactivation in FPA and Broca’s area may index a shared dysfunction in specific neurocognitive domains. The FPA is integral to integrating complex cognitive and emotional information during future-oriented thinking (44), while Broca’s area is involved in sequencing and inhibiting planned actions and internal verbal routines. Thus, their common hypoactivation could reflect a transdiagnostic deficit in the top-down regulation of internally generated, affect-laden cognition and action sequences, a process relevant to both obsessions/worries and compulsions.

### 4.2 OCD is more severely impaired in the DLPFC

This study demonstrates significantly greater DLPFC hypoactivation in OCD versus GAD, aligning with prior neuroimaging evidence showing DLPFC hyperactivation in OCD during cognitive tasks (45) and reduced functional connectivity between right DLPFC and orbitofrontal cortex (OFC) (46). The DLPFC’s pathophysiological role in OCD involves network dysregulation within CSTC circuits governing cognitive control (10) and impaired top-down modulation of limbic regions (e.g., anterior cingulate, OFC) (10, 34), further evidenced by therapeutic efficacy of repetitive transcranial magnetic stimulation (rTMS) targeting DLPFC for symptom amelioration (35, 36). These distinct neurofunctional impairments highlight a potential quantitative difference in the severity of impairment along a specific neurocognitive dimension between OCD and GAD. The DLPFC is a core substrate for top-down cognitive control and effortful regulation (37). Its pronounced hypoactivation in OCD suggests a particularly severe deficit in this “cognitive control” dimension, which may contribute to the perceived intrusiveness of obsessions and the compulsive drive. This finding does not merely justify diagnostic categories but identifies a graded neural marker for a dimension of dysfunction that is present in both disorders yet more severely expressed in OCD.

### 4.3 Reduced left Broca activation and its relationship with compulsive symptoms in OCD

We identified hypoactivation in left Broca’s area in OCD, with activation levels negatively correlating with Y-BOCS total and compulsion scores. To our knowledge, this is the first report of Broca’s area alterations in OCD. Beyond its traditional role in language, Broca’s area also subserves cognitive functions including working memory and cognitive control (38, 47), suggesting its involvement extends beyond language. Although not part of core CSTC circuits, Broca’s area resides within frontal networks implicated in OCD’s executive dysfunction. Compulsive behaviors are thought to arise, in part, from deficits in cognitive control and inhibition—functions subserved by these frontal networks (48). This may explain why its hypoactivation correlated with compulsions but not obsessions. A glutamate-GABA imbalance in frontal regions (e.g., anterior cingulate) (39) could indirectly influence Broca’s area via local neurotransmitter environments, given its glutamatergic/GABAergic neuronal density. Supporting its pathophysiological relevance, network analysis identifies Broca’s area as an epicenter in schizophrenia (49), highlighting its transdiagnostic potential. Thus, Broca’s area represents a novel target for OCD neuromodulation therapies and mechanistic studies. Its involvement, particularly the correlation with compulsion severity, underscores its role in a transdiagnostic circuit governing the inhibition of maladaptive action sequences. This aligns with network analyses identifying it as a hub in other disorders (e.g., schizophrenia), reinforcing its relevance beyond a single diagnostic category.

### 4.4 Evaluation of the potential diagnostic implications of fNIRS markers

Our ROC analysis indicated that fNIRS-derived integral values could differentiate combined patient groups (OCD and GAD) from healthy controls with moderate accuracy (e.g., AUC = 0.769 for right FPA), a level of accuracy comparable to that reported in studies proposing fNIRS as an adjunct diagnostic tool in psychiatry (40, 50). However, our study reveals a critical limitation that constrains this general promise. First, the reported AUC values are moderate and were derived from the same sample without external validation. More decisively, and in contrast to the general aim of disorder differentiation, no fNIRS marker in our study reliably differentiated OCD from GAD. This inability to achieve disorder-specific discrimination is essential for clinical diagnostic utility. Therefore, our findings must be interpreted as exploratory. Importantly, the inability of fNIRS markers to differentiate OCD from GAD, coupled with their ability to distinguish patients from HCs, challenges the pursuit of disorder-specific biomarkers. Instead, it supports the potential of fNIRS to quantify transdiagnostic, dimensional dysfunction in prefrontal systems related to cognitive-emotional integration and control. Future validation studies should test whether fNIRS-derived measures of these neural dimensions correlate with continuous behavioral phenotypes (e.g., cognitive flexibility, compulsivity) across diagnostic spectra, rather than solely aiming to classify discrete disorders.

In summary, our fNIRS findings are exploratory and hypothesis-generating. While prefrontal measures differentiated patients from controls at a group level, they cannot currently inform diagnosis, treatment selection, or prognosis. To assess potential future clinical relevance, studies must investigate (1): whether baseline fNIRS activity predicts response to therapy (e.g., CBT, medication) (2); how these measures relate to illness duration, severity, or complementary biological markers; and (3) if they could eventually help guide personalized neuromodulation targets. Until such studies are completed, these findings remain strictly research-oriented.

### 4.5 Limitations

Several limitations of the present study must be considered. First, and most critically, the near-universal use of SSRIs/SNRIs represents a profound confounder. These medications directly modulate neurovascular coupling—the physiological basis of the fNIRS signal. Evidence suggests that SSRI treatment itself can alter prefrontal executive network activity. Therefore, the observed hypoactivation likely reflects a composite of disease-specific traits, medication-induced normalization, and direct pharmacological effects. Importantly, based on our cross-sectional data, we cannot determine whether the medication acted to normalize prefrontal activity (thereby potentially attenuating group differences) or to induce its own atypical activation pattern (which could have accentuated apparent group differences). Our cross-sectional design cannot disentangle these influences. Second, the presence of comorbid depressive symptoms in a portion of the GAD cohort may have influenced the neurofunctional profile. Future studies would benefit from including a clinical comparator group with major depressive disorder to better isolate disorder-specific and transdiagnostic neural features. Third, our multiple comparison correction strategy (applying FDR within each hemodynamic parameter) did not control the family-wise error rate across the full set of ROI ×parameter tests conducted, which may increase the risk of false-positive findings. Consequently, the statistical findings reported in this study should be interpreted as exploratory and requiring replication in future studies that employ a pre-registered, hierarchical correction protocol (e.g., correcting across all omnibus tests first). Fourth, the limited sample size of this exploratory study, which was not based on a formal power calculation, constrains statistical power and generalizability. Future validation in larger cohorts, powered by the effects observed here, is required. Fifth, our analytical approach is limited. The pairwise group comparisons used, while directly testing group differences, are suboptimal for dissociating neural signals that are shared across disorders from those that are disorder-specific. For instance, planned contrasts such as combined OCD+GAD vs. controls (for shared effects) or OCD vs. (GAD+controls) (for OCD-specific patterns) would offer a more direct and powerful test of these distinct hypotheses. Future studies employing such targeted contrasts are needed to better delineate common and unique pathophysiology within the anxiety spectrum. Finally, the results of our exploratory ROC analysis are likely optimistically biased, as the input variables were pre-selected based on prior significant group differences. This may inflate the estimated classification accuracy; therefore, these findings require validation in independent cohorts using *a priori* defined metrics.

## 5 Conclusions

To our knowledge, this represents the first fNIRS study comparing VFT evoked responses across OCD, GAD, and HCs, revealing three key findings (1): Significant [oxy-Hb] hypoactivation in both disorders versus HCs, with OCD showing markedly greater reductions in left DLPFC and FPA (2); Negative correlations between left Broca’s area activation and obsessive-compulsive symptom severity (3); Diagnostic utility of regional integral values (DLPFC/FPA/Broca’s) for distinguishing patient groups from HCs, particularly right FPA integral (AUC = 0.769). These results elucidate transdiagnostic neurofunctional impairments while identifying disorder-specific pathophysiological profiles, advancing fNIRS-based biomarkers for differential diagnosis of anxiety-related disorders.

## Data Availability

The raw data supporting the conclusions of this article will be made available by the authors, without undue reservation.
